# Comparison between the Clancy Behavior Scale and the Modified Checklist for Autism in Toddlers in Taiwan

**DOI:** 10.3390/children11050557

**Published:** 2024-05-06

**Authors:** Ching-Lin Chu, Wen-So Su, Lai-Sang Iao, Chin-Chin Wu, Yuh-Ming Hou

**Affiliations:** 1 Department of Educational Psychology & Counseling, National Pingtung University, Pingtung 900391, Taiwan; clchu@mail.nptu.edu.tw; 2 Department of Psychology, Kaohsiung Medical University, Kaohsiung 807378, Taiwan; wsu@kmu.edu.tw; 3 Department of Medical Research, Kaohsiung Medical University Hospital, Kaohsiung 807377, Taiwan; 4 Department of Psychology, Nottingham Trent University, 50 Shakespeare Street, Nottingham NG1 4FQ, UK; 5 Department of Psychiatry, Ditmanson Medical Foundation Chia-Yi Christian Hospital, Chai-Yi 60002, Taiwan

**Keywords:** autism spectrum disorder, Clancy Behavior Scale, M-CHAT, sensitivity, specificity

## Abstract

(1) Background: Precise diagnosis and early intervention are crucial for toddlers with autism spectrum disorder (ASD) to achieve a better prognosis. This study investigated the efficacy of the Clancy Behavior Scale (CBS) and Modified Checklist for Autism in Toddlers (M-CHAT) in detecting ASD among toddlers under 30 months of age. (2) Methods: A total of 215 toddlers (117 with ASD and 98 with development delays) aged between 18 and 29 months participated in this study. All the primary caregivers of these toddlers were recruited to complete the CBS and M-CHAT. (3) Results: The findings indicated that the accuracy of the CBS and M-CHAT was promising, and the short forms of these two instruments performed better than their full versions. The CBS:9 critical items presented a sensitivity of 0.75 and a specificity of 0.74, while the M-CHAT:14 brief items showed a sensitivity of 0.72 and a specificity of 0.85. (4) Conclusions: The diagnostic accuracy of high-risk ASD toddlers improved via the combination of CBS and M-CHAT, particularly when the information gathered from these two instruments were consistent. The findings may provide implications for enhancing the early detection of ASD.

## 1. Introduction

Autism spectrum disorder (ASD) is known as a lifelong neurodevelopmental disorder characterized by deficits in social and communication skills that are accompanied by a restricted range of interests and repetitive behavior patterns [1]. Early diagnosis of ASD enables children and families to receive early intervention. Previous studies showed that early intervention can significantly improve the deficits and outcomes for children with ASD [2,3,4,5]. The importance of early detection and prompt diagnosis is accentuated by the benefits of early intervention. However, children with ASD are usually diagnosed after age 3 [6,7], impacting on the timing of access to early intervention. Thus, expediting the early detection and early diagnosis of children with ASD is crucial.

The prevalence of diagnosis of ASD in children has escalated substantially. Recent studies have revealed that the estimated prevalence is at least 1.5% [8,9,10,11]. However, when compared to Western countries, Taiwan has a relatively lower prevalence of ASD, especially for young children [12,13,14]. This circumstance is mainly caused by some factors, such as the lack of proper screening tools for toddlers, and inadequate training and experience among clinicians in screening or diagnosing ASD in young children [15]. Hence, there is a compelling need to enhance the early screening and diagnosis of ASD in Taiwan. Yet, given their heavy workloads, it is difficult for clinicians in Taiwan to acquire adequate training in using diagnostic tools, namely the Autism Diagnostic Interview-Revised (ADI-R) [16], Autism Diagnostic Observation Schedule (ADOS) [15], and ADOS-2 [17], not to mention the shared attribute of these instruments being time-consuming to administer. Thus, a concise and easily administered screening measure is required.

The Screening Tool for Autism in Two-Year-Olds (STAT) [18] is an interactive screening tool designed to detect ASD in high-risk samples. Like the ADOS, training and certification are required before one can administer the STAT. To date, there are 120 clinicians and researchers worldwide who have received certification. Wu et al. [19], in a recent study, explored the validity of the STAT when used on toddlers under 24 months of age. They recruited 57 toddlers with ASD and 62 toddlers with developmental delays (DD) aged between 16 and 24 months (Time 1), finalizing their diagnoses at 18 months after Time 1. Their found that the STAT demonstrated good accuracy (>0.80). Nowadays, the Taiwanese version of the Screening Tool for Autism in Two-Year-Olds (T-STAT) [20] has been developed with good accuracy in detecting ASD in young children prior to 36 months of age [15,21], but it can be only used in research at this stage.

As mentioned above, clinicians in Taiwan, due to their tight schedules, often prefer parent-report screening tools that can be quickly completed but remain valid in identifying ASD in young children. The most frequently chosen instruments are the Clancy Behavior Scale (CBS) [21] and Modified Checklist for Autism in Toddlers (M-CHAT) [22], and especially the former is the first ASD screening tool available in Taiwan. Yet, Sun et al. [21] addressed that when they utilized the CBS to detect ASD in school-age children, it demonstrated good specificity but poor sensitivity. Hsieh et al. [23] recruited 147 children under 16 years old, including 49 with ASD and 98 with other developmental disabilities. They used 14 as the cutoff score for the CBS, and at the end it showed good sensitivity (0.84) but poor specificity (0.60). Both studies revealed that the CBS has not reached a balance between sensitivity and specificity when applied to identify ASD in school-age children.

To explore the effectiveness of the CBS on younger children in Taiwan, Wu et al. [24] recruited participants aged between 18 and 47 months, comprising 62 children with ASD and 90 children with DD. Wu and his colleagues used the same cutoff score as that of Hsieh et al. [23], and the CBS showed poor sensitivity (0.61) but good specificity (0.87). When lowering the cutoff score from 14 to 12, the CBS demonstrated a sensitivity of 0.74 and a specificity of 0.73, representing fair accuracy. In addition, through discriminant analysis, 9 out of the 14 CBS items were selected as critical items. Wu et al. adopted 7 as the cutoff score and found a sensitivity of 0.76 and a specificity of 0.76, suggesting that the CBS, while adopting a lower cutoff threshold, could be effective in detecting ASD in children under 48 months of age with fair accuracy.

The M-CHAT is an instrument that has been widely studied and used for ASD screening purposes. It comprises 23 yes/no questions created to evaluate toddlers aged between 16 and 30 months. Toddlers who fail to pass any 3 out of these 23 items, or any 2 out of the 6 M-CHAT critical items are considered at high risk for ASD or DD. In Taiwan, Wong et al. [25] recruited 236 children (113 with ASD and 123 with DD) aged between 18 and 47 months as their participants and found fair sensitivity (0.77) and specificity (0.72) when classifying those who failed on any 4 out of the 23 M-CHAT items as children with ASD. Wong et al. also selected 14 M-CHAT items as critical questions to develop the ‘‘Brief 14’’. They later reported fair sensitivity (0.71) and good specificity (0.82) when identifying participants who failed on any 3 of the Brief 14 items as children with ASD. Wong et al.’s findings are not only consistent with those in the previous studies [26], but also illustrated that the M-CHAT needs modifications when utilized in a different culture. Recently, a revised version of the M-CHAT (M−CHAT-R/F) with good psychometric properties for detecting ASD in young children was reported by some researchers from Taiwan [27], but currently the M-CHAT is still the primary ASD screening tool administered by clinicians in Taiwan. Thus, the M-CHAT was chosen as a research measure in this study.

The early detection and prompt diagnosis of ASD are decisive for connecting toddlers with early intervention and treatment. Previous studies (e.g., [2,3]) show that early intervention for toddlers with ASD can result in better outcomes, especially in improving their cognitive abilities, language skills, and autistic symptoms. In Taiwan, both the CBS and M-CHAT are commonly used for ASD screening but have not been fully studied. The shortage of tools with robust validity undermines the effectiveness of ASD screening, consequently jeopardizing the early intervention efforts for young children with ASD. Given this, the present study aimed to assess and compare the effectiveness of both the CBS and M-CHAT in detecting ASD in toddlers under 30 months of age. That is, the goal of this study was to enhance early ASD screening practices, and to achieve this, the following questions were investigated:

(1) What differentiates toddlers with ASD from those with DD according to their performances on the CBS and M-CHAT? In addition, are there any differences between the full and short forms of these two screening tools?
(2) How do the CBS and M-CHAT perform in terms of sensitivity and specificity in screening for ASD?
(3) What is the accuracy of the CBS and M-CHAT in detecting ASD in toddlers prior to 30 months of age, and how do they compare?

## 2. Materials and Methods

### 2.1. Participants

This study included 215 toddlers (117 with ASD and 98 with DD) aged between 18 and 29 months. None of them had sensory or motor impairments or a history of any genetic disorders. Participants’ diagnoses were made by a multidisciplinary team along with the Diagnostic and Statistical Manual of Mental Disorders, Fifth Edition, Text Revision (DSM-5-TR) [1]. As per the DSM-5-TR criteria for ASD, a child must exhibit at least three deficits in social communication/interaction skills and two restricted/repetitive behaviors. All toddlers with ASD went through evaluations based on their developmental history, caregivers’ reports, cognitive and adaptive functioning assessments, behavioral observations, and the results of the ADOS [15]. Toddlers who scored below 85 on the Mullen Scales of Early Learning (MSEL) [28] or below a T-score of 35 on any of the four cognitive scales (i.e., visual reception, fine motor, receptive language, and expressive language) were categorized as children with DD if they did not meet the DSM-5-TR criteria for ASD.

The mental ages (MAs) of these toddlers were calculated by averaging the age equivalents of the four cognitive scales from the MSEL. The independent-samples t tests were performed to compare the chronological age, MAs, and ADOS scores between the ASD and the DD groups. Chi-square tests were also conducted to analyze the gender ratio and parents’ education level in these two groups. Table 1 presents the demographic characteristics of the participants.

### 2.2. Procedures

The current study received ethical approval from the Ditmanson Medical Foundation Chia-Yi Christian Hospital Research Ethics Committee and the Kaohsiung Medical University Chung-Ho Memorial Hospital Institutional Review Board. All participants were referred to this study by their treating clinicians. Before the assessment began, the participants’ parents signed the informed consents and then completed both the CBS and M-CHAT, while their children were assessed using the MSEL [28] and the ADOS [29]. The ADOS were administered by two of the authors, who received research training and certification in Taiwan under the supervision of Dr. Catherine Rice’s team.

### 2.3. Measures

#### 2.3.1. Clancy Behavior Scale (CBS) [21,24]

The CBS, a parent-report questionnaire, was used to detect ASD in children under four. This scale comprises 14 items, designed to evaluate whether a child can perform behaviors that are typical of his/her age. That is, each examinee is assessed according to how frequently they perform the behaviors described in the CBS items, with “Never” (score of 0), “Occasionally” (score of 1), and “Usually” (score of 2). When adopting 12 as a cutoff score, the CBS showed fair sensitivity (0.74) and specificity (0.73) [24]. Additionally, 9 CBS items were selected as critical items, including item 1 (great difficulty playing with other children), 2 (acts as deaf), 7 (laughing for no apparent reason), 8 (not cuddly as a baby), 10 (no eye contact), 11 (unusual attachment), 12 (spins objects), 13 (repetitive and sustained odd play), and 14 (standoffish manner). To achieve fair sensitivity (0.76) and specificity (0.76), adopting 7 as a threshold is recommended while using these 9 critical items.

#### 2.3.2. Modified Checklist for Autism in Toddlers [22,25]

The M-CHAT consists of 23 yes/no questions designed to detect ASD in toddlers. Under the original criteria, a toddler is identified as high-risk for ASD if they fail to pass any 3 out of these 23 items or any 2 out of the 6 M-CHAT critical items. However, in Taiwan, Wong et al. [25] proposed alternative criteria for identifying ASD. According to their findings, a toddler is considered high-risk for ASD if they fail any 4 out of the 23 items or fail 3 out of the 14 brief M-CHAT items. These 14 brief items include: interest in other children (item 2), pretend play (item 5), pointing for requesting (item 6), pointing for interest (item 7), showing (item 9), imitation (item 13), response to name (item 14), following pointing (item 15), following gaze (item 17), attract attention (item 19), suspected deafness (item 20), language comprehension (item 21), wandering without purpose (item 22), and social referencing (item 23).

#### 2.3.3. Mullen Scales of Early Learning (MSEL) [28]

The MSEL [28] is a standardized comprehensive developmental test invented to calculate the MAs for preschool children aged between 0 and 68 months. The MSEL consists of four subscales (i.e., visual reception, fine motor, receptive language, and expressive language) that produce T-scores with a mean of 50 and can be applied to determine a composite score, indicative of early learning, with a mean of 100. In this study, an overall MAs was derived for each participant by averaging the age equivalents obtained from the aforementioned four scales. The Taiwan version of the MSEL has been employed to evaluate toddlers and demonstrated a moderate to strong correlation with the Vineland Adaptive Behavior Scale-Chinese Version. Further, it displays excellent internal consistency and interrater reliability [30].

#### 2.3.4. Autism Diagnostic Observation Schedule (ADOS) [29]

The ADOS is a semi-structured, play-based observational tool that comprises four modules, each chosen and administered based on a child’s age and expressive language. Both the ADOS and the ADI-R are treated as gold-standard instruments for diagnosing ASD [16]. The former provides a standardized opportunity to observe and assess communication, reciprocal social interaction, stereotypic behaviors, and restricted interests. Each module of the ADOS includes an algorithm with cutoffs that allow users to categorize examinees into three different groups: autism, autism spectrum (i.e., pervasive developmental disorder-not otherwise specified (PDD-NOS)), or non-ASD. The original algorithm of the ADOS requires a test taker to meet the thresholds for the communication domain (COM), the social interaction domain (SOC), and the combined domains of COM and SOC (COMSOC) for classification purposes. In the present study, each participant was assessed using the Chinese version of the ADOS authorized by the publisher (WPS). This version employs the same cutoffs as those adopted by the original ADOS and has demonstrated good validity [19]. Its sensitivity and specificity in this study, when compared with clinical diagnosis, were 1 and 0.94, respectively. All toddlers in this study went through assessment using ADOS module one.

### 2.4. Statistical Analyses

Statistical analyses were performed using IBM SPSS 21. Concurrent validity was assessed by calculating Pearson correlations among the CBS, the M-CHAT, and the ADOS domains (i.e., COM, SOC, COMSOC). Moreover, the independent samples t tests were conducted to examine the differences between the ASD and the DD groups on the CBS and the M-CHAT. Since these two groups were unmatched in terms of their MAs, analysis of covariance (ANCOVA) was further conducted to analyze the performance differences between these two groups. Additionally, Spearman’s rho tests were employed to explore the correlations among the CBS, M-CHAT, and ADOS. Furthermore, Receiver Operating Characteristics (ROC) area-under-curve (AUC) was used to examine the accuracy of determining the optimal range for the two screening measures.

## 3. Results

The concurrent validity of the CBS and M-CHAT tools was analyzed through Pearson correlations with the ADOS (Table 2). The scores which participants received from the CBS:Full items were moderately correlated with those they obtained on the ADOS COM (r = 0.46) and COMSOC (r = 0.48), and were also highly correlated with their scores on the ADOS SOC score (r = 0.51). Likewise, the participants’ scores on the CBS:9 critical items demonstrated high correlations with those that they had on the ADOS COM (r = 0.53), SOC (r = 0.60), and COMSOC (r = 0.59). In addition, the scores obtained by the participants on the M-CHAT:Full items manifested high correlations with their scores on the ADOS COM (r = 0.51), SOC (r = 0.58), and COMSOC (r = 0.57), while their achievement on the M-CHAT:14 brief items showed high correlations with their performance on the COM (r = 0.54), SOC (r = 0.61), and COMSOC (r = 0.60) on the ADOS.

The differences between the ASD and the DD groups on the CBS and M-CHAT are shown in Table 3, indicating that the ASD group performed significantly differently on both the CBS and M-CHAT from the DD group. That being said, toddlers with ASD, when compared to those with DD, received higher scores on the CBS and M-CHAT. Given that these two groups differed in their MAs, ANCOVA was conducted, and the statistical outc omes remained unchanged (Table 4).

**Figure 1:**
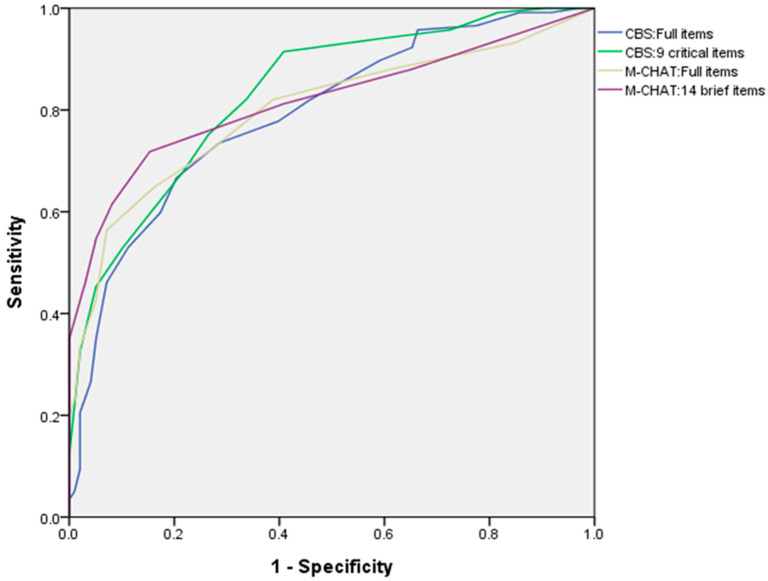
Receiver Operating Characteristics curves for the CBS and M-CHAT. CBS = Clancy Behavior Scale; M-CHAT = Modified Checklist for Autism in Toddlers.

The ROC analyses showed that the AUC of the CBS:Full items and CBS:9 critical items were 0.79 (confidence interval = 0.73–0.85) and 0.83 (confidence interval = 0.78–0.89), respectively. The effect size (d) was 1.39 for CBS:9 and 1.16 for CBS:Full. In addition, the AUC of the M-CHAT:Full items and M-CHAT:14 brief items were 0.80 (confidence interval = 0.74–0.86) and 0.82 (confidence interval = 0.76–0.87), respectively (see Figure 1 and Table 5).

Sensitivity and specificity, while adopting different cutoff scores, were also calculated to assess the accuracy of the CBS and M-CHAT (both full and short forms) in screening ASD. As shown in Table 5, the CBS:Full items, for instance, was found to have fair sensitivity (0.74) and specificity (0.71) when using 12 as a cutoff score. To be more precise, among 117 toddlers with ASD, 86 failed to pass 12 or more items on the full version of the CBS. Yet, among another 98 toddlers who did not have ASD, 70 of them failed fewer than 12 items on the same screening tool. When lowering the cutoff score from 12 to 7, the CBS:9 critical items displayed fair sensitivity (0.75) and specificity (0.74). On the other hand, the M-CHAT:Full items showed fair sensitivity (0.72) and specificity (0.74) when using 4 as a cutoff score. Regarding the M-CHAT:14 brief items, it demonstrated fair sensitivity (0.72) and good specificity (0.85) with a cutoff score of 3 (see Table 5). It is worth noting that combining the CBS and M-CHAT resulted in better sensitivity but poor specificity (see Table 6).

## 4. Discussion

Timely diagnosis is crucial for young children with ASD because it can expedite the process of early intervention that improves treatment outcomes. Like the trends reported in other countries, Gau et al. [31] also noticed that there was a growing awareness of early indicators of ASD in Taiwan. However, children with ASD in Taiwan, on average, receive their diagnoses around the age of 4.5 years, along with a relatively lower prevalence when compared to Western countries [32]. It is believed that these circumstances are mainly caused by the shortage of appropriate ASD-specific screening tools for young children. Thus, there is an urgent need to develop proper instruments for young children with ASD in Taiwan. In addition, the diagnostic criteria for ASD were significantly changed for the DSM-5-TR, but most studies [21,24] that examined the effectiveness of the CBS and M-CHAT relied on early versions of DSM. Therefore, this study, according to the DSM-5-TR criteria, aimed to explore the accuracy of these two screening tools in detecting ASD among toddlers in a clinical setting in Taiwan.

Regarding the concurrent validity of the CBS and M-CHAT, our findings revealed that the correlations between CBS:Full items scores and ADOS scores, as well as those between CBS:9 critical items scores and ADOS scores, ranged from moderate to high. The CBS:9 critical items showed slightly better concurrent validity compared to the CBS:Full items. In addition, the correlations between M-CHAT scores (both full items and 14 brief items) and ADOS scores appeared high. The M-CHAT:14 brief items showed slightly better concurrent validity than the M-CHAT:Full items. Consistent with previous research results [21,25], younger children with ASD in Taiwan obtained higher scores on both the CBS and M-CHAT. The findings also showed that the CBS:9 critical items had a slightly superior effect size (d = 1.39) compared to the CBS:Full items (d = 1.16). Similarly, the M-CHAT:14 brief items had a slightly greater effect size (d = 1.34) than the M-CHAT:Full items (d = 1.22). It appears that the short forms of both the CBS and M-CHAT, the versions that excluded items with insufficient discriminative power, demonstrated better psychometrics properties in Taiwan. Overall, the CBS and M-CHAT, both full and short forms, exhibited the potential to distinguish young children with ASD from those with DD.

In terms of the utility of the CBS, our findings revealed that the CBS:Full items had fair sensitivity (0.74) and specificity (0.71) with a cutoff score of 12. It successfully detected 86 toddlers with ASD and 70 toddlers with DD among of 215 young children. The overall accuracy rate of the CBS:Full items in identifying toddlers with ASD was 0.73. When adopting 7 as the threshold, the CBS:9 critical items demonstrated a sensitivity of 0.75 and a specificity of 0.74. In this study, the CBS:9 critical items correctly identified 88 toddlers with ASD and 72 toddlers with DD out of 215 young children. The overall accuracy rate of the CBS:9 critical items was 0.74. Moreover, the data shown by the AUC exhibited that the CBS:9 critical items demonstrated better accuracy than the CBS:Full items. In line with previous studies [21,24], the CBS could be employed to identify young children with ASD under the age of 4 years old. The CBS was initially curated to distinguish children displaying severe or traditional forms of autism, utilizing a more specific diagnostic framework as opposed to the wider scope of the ASD diagnosis [21] and has been used in Taiwan for approximately 40 years. Although the CBS does not incorporate the latest diagnostic criteria for ASD [21], our findings suggest that it can still be applied to detect young children with ASD.

Similar to the performance of the CBS, the M-CHAT:Full items demonstrated a sensitivity of 0.72 and a specificity of 0.74 with a cutoff score of 4, indicating that it accurately identified 84 toddlers with ASD and 72 toddlers with DD among 215 young children. The overall accuracy rate of the M-CHAT:Full items was 0.73. When adopting a cutoff score of 3, the M-HAT:14 brief items showed a sensitivity of 0.72 and a specificity of 0.85. It successfully detected 84 toddlers with ASD and 83 toddlers with DD from 215 young children. The overall accuracy rate of the M-CHAT:14 brief items was 0.78. As reported in the previous studies [25], the M-CHAT could be applied to identify young children with ASD in Taiwan. In addition, the AUC measures indicated that the M-CHAT:14 brief items appeared to perform better than the M-CHAT:Full items regarding ASD identification. Thus, the M-CHAT:14 brief items would be recommended for ASD screening purposes. In general, since the M-CHAT was initially developed to detect ASD in toddlers, all the findings mentioned above seem convincing. Our results support those reported in previous research, implying that one must take cultural differences into account before using the M-CHAT [26]. In Taiwan, the parents of children at high risk for ASD tend to assign lower scores on the M-CHAT compared to Western parents [33], because certain behaviors might be considered acceptable or tolerable in Taiwan, and this might lead to an underestimation of ASD symptom severity. Further, a low M-CHAT score could also manifest a social desirability bias. Many parents would underreport their children’s ASD symptoms to avoid social stigma.

Contrary to our expectation, combining both the CBS:Full items and M-CHAT:Full items positive did not result in better diagnostic accuracy. It did improve the sensitivity (0.88) but compromised the specificity (0.53). Similar results (sensitivity = 0.89, specificity = 0.63) were found when combining both the CBS:9 critical items or M-CHAT:14 brief items positive. That is, an optimal balance between sensitivity and specificity could not be attained with the combination of the CBS and M-CHAT in this study. However, the short form showed high sensitivity and could exactly detect DD in toddlers at a rate of above 63%. These findings imply that combining the short forms of these two instruments might be useful to distinguish toddlers with ASD from those with DD.

It is noteworthy that the combination of the CBS and M-CHAT used to detect ASD in toddlers would result in false positive rates (36.7–46.9%). These results, as noticed by some researchers [34], might be caused by parents’ concerns about their child’s development. Each participant in this study, who was suspected of having developmental disabilities by their parents, was recruited from a hospital. Accordingly, the high false positive rate was observed. However, the high false positive rate in this study does not necessarily imply a flaw in the CBS or M-CHAT. More precisely, this phenomenon may simply indicate that parents have perceived their children to have ASD features, and these toddlers need to be followed up carefully and longitudinally. More importantly, there were 13–14 toddlers with ASD who were not identified by these two screening tools, and this may be attributed to their parents’ under-reporting, misunderstanding, or denial of their children’s ASD features, which illustrates the need for parents to receive information or education about the early signs of ASD.

Additionally, when a positive outcome was shown on both the short forms of the CBS and M-CHAT, it meant that 68 out of 73 (93.15%) toddlers with developmental disabilities received the diagnosis of ASD. Conversely, when the full forms of these two screening tools were utilized and yielded a negative outcome, it meant that 62 out of 75 (82.67%) toddlers with developmental disabilities received the diagnosis of DD. Overall, the accuracy of diagnosing ASD or DD in this study was promising. Past studies [35] claimed that the ASD diagnostic accuracy for young children might be compromised when incorporating information gathered from a parent-report scale (i.e., the ADI-R) with that from a behavioral observation method (i.e., the ADOS), but our findings differed from those from previous research, that is, information provided in a parent-report questionnaire, as long as it is consistent, can still help examiners reliably classify toddlers with ASD and those with DD, implying an alternative to detect ASD in toddlers.

### Limitations

In Taiwan, both the CBS and M-CHAT are commonly used to identify ASD. The findings of this study revealed that these two screening tools demonstrated similar efficacy in detecting ASD among toddlers from a rural area in Taiwan. However, this study has some limitations: First, given the discrepancies in the prevalence of ASD between urban and rural areas [32,36], it is necessary to further examine the accuracy of the CBS and M-CHAT in detecting ASD in urban areas. Secondly, the ASD screening in this study relied solely on the information gathered from parent-report questionnaires and did not incorporate follow-up interviews, which are beneficial assessment methods. Thus, it is recommended that future studies examine whether the effectiveness of ASD screening would be improved with follow-up interviews. The same suggestion also applies to observation-based screening tools such as STAT. Thirdly, this study adopted a cross-sectional design. Past studies suggested that the early detection of ASD needs to be echoed [37,38,39]. Thus, a longitudinal research design is preferred in a future study examining the stability of the ASD screening instruments. Moreover, one of the challenges encountered in this study while identifying and diagnosing ASD was the difficulty to attain optimal sensitivity and specificity in the research measures, particularly when female toddlers were assessed. Hence, there is a pressing need for future research to focus on developing a screening tool that can accurately and effectively diagnose ASD in both genders. More importantly, the factors (e.g., cognitive levels) that might affect the detection of ASD in different populations need to be taken into account as well.

## 5. Conclusions

Early diagnosis and prompt intervention are critical for young children with ASD and their families. Due to the greater brain plasticity in infancy, early intervention provides an opportunity to modify the trajectory of ASD [40]. This highlights the importance of the early detection and timely diagnosis of ASD. The reliable diagnosis of young children with ASD can be made via the combination of parent-report scales and observation-based tools [35]. In contrast to previous findings, two screening tools, the CBS and M-CHAT, were employed to detect ASD in toddlers in this study, and the results revealed that the accuracy of these two instruments in identifying toddlers with ASD ranged from fair to high, the latter being the case especially when the data that they provided appeared to be consistent. Most notably, the short forms of both the M-CHAT:14 and CBS:9 showed better efficacy in ASD screening compared to their full versions. Further, M-CHAT:14 may offer additional advantages over CBS:9 in the screening processes. In general, these findings provide implications for the clinicians in Taiwan when assessing or diagnosing ASD.

## Figures and Tables

**Table 1:** Demographic characteristics of all participants (*n* = 215).

Variable	ASD (*n* = 117)	DD (*n* = 98)	*p*
CA (months)			
Mean (SD)	24.78 (2.99)	22.79 (3.26)	<0.001
MAs (months)			
Mean (SD)	14.75 (3.85)	17.80 (3.82)	<0.001
ADOS total scores			
Mean (SD)	18.03 (2.72)	3.54 (1.95)	<0.001
Gender Ratio			
Male: Female	104:13	69:29	0.001
Mother’s education level			
Senior high or lower	40	36	0.864
College	64	50	
Postgraduate	13	12	
Father’s education level			
Senior high or lower	38	47	0.056
College	63	41	
Postgraduate	16	9	
Missing	0	1	

ASD = autism spectrum disorder; DD = developmental delays; CA = chronological age; MAs = mental ages; ADOS = Autism Diagnostic Observation Schedule.

**Table 2:** Concurrent validity among the ADOS, CBS, and M-CHAT.

	ADOS		
	COM	SOC	COMSOC
CBS:Full items	0.46 ***	0.51 ***	0.51 ***
CBS:9 critical items	0.53 ***	0.60 ***	0.59 ***
M-CHAT:Full items	0.51 ***	0.58 ***	0.57 ***
M-CHAT:14 brief items	0.54 ***	0.61 ***	0.60 ***

ADOS = Autism Diagnostic Observation Schedule; COM = communication; SOC = social interaction domain; COMSOC = COM + SOC; CBS = Clancy Behavior Scale; M-CHAT = Modified Checklist for Autism in Toddlers. *** *p* < 0.001.

**Table 3:** Performance of the screening tools in the ASD and DD groups.

	ASD (*n* = 117)	DD (*n* = 98)	*p*
CBS:Full items			
Mean (SD)	14.35 (4.45)	9.26 (4.30)	<0.001
CBS:9 critical items			
Mean (SD)	8.84 (3.29)	4.45 (2.97)	<0.001
M-CHAT:Full items			
Mean (SD)	6.53 (4.04)	2.49 (2.17)	<0.001
M-CHAT:14 brief items			
Mean (SD)	5.16 (3.59)	1.38 (1.46)	<0.001

CBS = Clancy Behavior Scale; M-CHAT = Modified Checklist for Autism in Toddlers; ASD = autism spectrum disorder; DD = developmental delays.

**Table 4:** Adjusted performance of the screening tools in the ASD and DD groups.

	ASD (*n* = 117)	DD (*n* = 98)	*p*
CBS:Full items			
Mean (SD)	14.35 (0.42)	9.26 (0.46)	<0.001
CBS:9 critical items			
Mean (SD)	8.78 (0.30)	4.51 (0.33)	<0.001
M-CHAT:Full items			
Mean (SD)	6.22 (0.31)	2.86 (0.34)	<0.001
M-CHAT:14 brief items			
Mean (SD)	4.86 (0.26)	1.74 (0.29)	<0.001

CBS = Clancy Behavior Scale; M-CHAT = Modified Checklist for Autism in Toddlers; ASD = autism spectrum disorder; DD = developmental delays.

**Table 5:** Sensitivity, Specificity, and AUC of the CBS and M-CHAT.

Screening Tools	Cutoff	Sensitivity	Specificity	AUC
CBS:Full item	12	86/117 (0.74)	70/98 (0.71)	0.792
CBS:9 critical item	7	88/117 (0.75)	72/98 (0.74)	0.833
M-CHAT:Full items	4	84/117 (0.72)	72/98 (0.74)	0.798
M-CHAT:14 brief items	3	84/117 (0.72)	83/98 (0.85)	0.817

CBS = Clancy Behavior Scale; M-CHAT = Modified Checklist for Autism in Toddlers; AUC = area under curve.

**Table 6:** Classification using the CBS and M-CHAT in two groups.

Full Form	ASD (*n* = 117)	DD (*n* = 98)
CBS (P) & M-CHAT(P)	67 (57.3%)	8 (8.2%)
CBS (P) & M-CHAT (N)	19 (16.2%)	20 (20.4%)
CBS (N) & M-CHAT (P)	17 (14.5%)	18 (18.4%)
CBS (N) & M-CHAT (N)	14 (12.0%)	52 (53.1%)
**Short Form**		
CBS (P) & M-CHAT(P)	68 (58.1%)	5 (5.1%)
CBS (P) & M-CHAT (N)	20 (17.1%)	21 (21.4%)
CBS (N) & M-CHAT (P)	16 (13.7%)	10 (10.2%)
CBS (N) & M-CHAT (N)	13 (11.1%)	62 (63.3%)

P = Positive; N = Negative; ASD = autism spectrum disorder; DD = developmental delays.

## Data Availability

The data presented in this study are available on request from the corresponding author. The data are not publicly available, because of ethical concerns.
